# Evaluation of Gustatory Function in Oral Submucous Fibrosis Patients and Gutka Chewers

**DOI:** 10.31557/APJCP.2019.20.2.569

**Published:** 2019

**Authors:** Balaji Babu Bangi, Uday Ginjupally, Lakshmi Kavitha Nadendla, Mounika Reddy Mekala, Jaya Lakshmi B, Aswani Kakumani

**Affiliations:** *Department of Oral Medicine and Radiology, Kamineni Institute of Dental Sciences, Narketpally, Telangana, India. *

**Keywords:** Oral submucous fibrosis, taste, tongue

## Abstract

**Aim:**

The aim of the study is to assess and compare taste perception among Oral submucous fibrosis (OSMF) patients, Gutka chewers without OSMF and healthy subjects.

**Materials and methods:**

Ninety subjects (30 OSMF, 30 Gutka chewers without OSMF and 30 controls) were enrolled in the study for assessing taste perception by filter paper strips impregnated with different taste qualities. Taste perception assessment was also done in stage I, II and III OSMF subjects. The obtained data were analyzed using SPSS 20.0 software.

**Results:**

The gustatory defect was related to sweet, sour, bitter and salt, with significant changes in sour (33.3% showed hypoguesia) taste in OSMF subjects and 13.3%showed hypoguesia to all tastants in gutka chewers and hypoguesia to salt, sour and bitter to grade III compared in grade I and II.

**Conclusion:**

This study proved that there is significant alterations to taste perception with sour, salt, and bitter and then to sweet in OSMF subjects.

## Introduction

Oral submucous fibrosis (OSMF) is a chronic, progressive, potentially malignant diseaseof the oral mucosa which was first described by Schwartz in 1952 under the term atropicaidiopathica (tropica) mucosae oris. Later In 1953, Joshi described this condition as ‘submucousfibrosis’. In 1966, Pindborg defined this disease as “an insidious chronic disease affecting any part of the oral cavity and sometimes pharynx. Although occasionally preceded by and/or associated with vesicle formation, it is always associated with juxtaepithelial inflammatory reaction followed by fibroelastic changes in the lamina propria, with epithelial atrophy leading to stiffness of the oral mucosa causing trismus and difficulty in eating.” Persons most commonly affected are Asians, and maximum number of cases has been reported in Indians with a prevalence ranging up to 0.4% (Rajendran, 1994; Wollina et al., 2015).

Etiology is multifactorial and most common factor for OSMF being arecanut. The factors responsible for fibrosis in areca nut are alkaloids, flavonoids and other trace elements like copper. Patients complain of burning sensation while having hot and spicy food. The fibrosis of mucosa also leads to difficulty in mastication, speech, and swallowing and pain in throat and ears and loss of gustation (Tak et al., 2014; Stillman et al., 2003).

Taste is a sensation which is perceived due to the presence of taste buds over the dorsum of tongue, larynx and oesophagus. Saliva which is a critical fluid in the oral cavity plays primary role in dissolving the taste stimulus to taste buds. Alteration in the taste perception can be due to any change in salivary flow. It is well known that taste perception influences food intake. After ingestion, gustatory receptors relay sensory signals to the brain, which segregates, evaluates, and distinguishes the stimuli, leading to the experience known as “flavor.” They are five taste qualities like sweet, salty, bitter, sour, and umami can be perceived by animals. Changes in these taste qualities may lead to altered food preferences and reduced appetite so it should be an important focus of attention in the treatment of patients in order to improve their nutritional status and the efficacy of the treatment (Hillary, 2015; NHANES, 2013). As the taste perception affects the nutrition which in turn affects the immune surveillance, that favors the transformation of premalignant disorders to oral cancer, and less importance was given in previous studies on taste dysfunction and its role in OSMF, so present study was undertaken 

## Materials and Methods

A comparative study was conducted for a period of 1 year to assess and compare taste alterations among ninety subjects between the age group of 20–45 years attending department of Oral Medicine and Radiology, Kamineni institute of dental sciences, Telangana. Institutional ethical committee approval has been taken (KIDS/ICE/2016/11). A specially designed proforma was constructed for interviewing the patient and informed consent was obtained for the patient participation in the study. The subjects were diagnosed with OSMF were considered and classified them based on the Khanna et al., (1995) classification (Berwal et al., 2014; More et al., 2012). Gutka chewers considered in the study had habit history of at least 6monthsand control group comprised apparently healthy subjects with no history of habits. Subjects those above the age of 45 years and patients undergoing treatment for OSMF and subjects with known systemic illness or those taking medications which may cause alteration in perception of taste were excluded from the study.

**Table1:** Gustatory Changes for Sweet and Sour Tastants Groups

Taste		Group			Total	*P-value*
		CONTROLS	GUTKA	OSMF		
	Dysguesia	1 (3.3%)	0 (0%)	0 (0%)	1 (1.1%)	
Sweet	Hyperguesia	2 (6.7%)	0 (0%)	1 (3.3%)	3 (3.3%)	0.127
	Hypoguesia	0 (0%)	4 (13.3%)	6 (20.0%)	10 (11.1%	
	Normal	27 (90%)	26 (86.7%)	23 (76.6%)	76 (84.4%)	
	Total	30 (100%)	30 (100%)	30 (100%)	90 (100%)	
	hyperguesia	3 (10%)	0 (0%)	1 (3.3%)	4 (4.4%)	
Sour	hypoguesia	0 (0%)	4 (13.3%)	10 (33.3%)	14 (15.6%)	
	normal	27 (90.0%)	26 (86.7%)	19 (63.3%)	72 (80.0%)	0.03*
	Total	30 (100%)	30 (100%)	30 (100%)	90 (100%)	

*, P value less than 0.05 is statistically significant

**Table 2:** Gustatory Changes for Salt and Bitter Tastants Groups

Taste		Group			Total	*P-value*
		CONTROLS	GUTKA	OSMF		
	Dysguesia	0 (0%)	0 (0%)	1 (3.3%)	1 (1.1%)	
Salt	Hyperguesia	0 (0%)	0 (0%)	1 (3.3%)	1 (1.1%)	
	Hypoguesia	1 (3.3%)	4 (13.3%)	3 (10%)	8 (8.9%	0.415
	Normal	29 (96.7%)	26 (86.7%)	25 (83.3%)	80 (89.9%)	
	Total	30 (100%)	30 (100%)	30 (100%)	90 (100%)	
	Dysguesia	0 (0%)	0 (0%)	1 (3.3%)	1 (1.1%)	
Bitter	Hyperguesia	0 (0%)	1 (3.3%)	0 (3.3%)	1 (1.1%)	
	Hypoguesia	1 (3.3%)	4 (13.3%)	4 (13.3%)	8 (8.9%)	
	Normal	29 (96.7%)	25 (83.3%)	25 (83.3%)	80 (88.9%)	0.41
	Total	30 (100%)	30 (100%)	30 (100%)	90 (100%)	

*, P value less than 0.05 is statistically significant

**Table 3:** Gustatory Changes for Sweet and Sour in Different Grades of OSMF

Taste		Grade			Total	*P-value*
		I	II	III		
	Hyperguesia	0 (0%)	1 (100%)	0 (0%)	1 (100%)	
	Hypoguesia	0 (0%)	5 (83.3%)	1 (16.7%)	6 (100%)	
Sweet	Normal	12 (52.2%)	6 (26.1%)	5 (21.7%)	23 (100%)	0.68
	Total	12 (40%)	12 (40%)	6 (20%)	30 (100%)	
	Hyperguesia	1 (100%)	0 (0%)	0 (0%)	1 (100%)	
Sour	Hypoguesia	4 (40%)	1 (10%)	5 (50%)	10 (100%)	0.018*
	Normal	7 (36.8%)	11 (57.9%)	1 (5.3%)	19 (100%)	
	Total	12 (40%)	12 (40%)	6 (20%)	30 (100%)	

*, P value less than 0.05 is statistically significant

**Table 4:** Gustatory Changes for Salt and Bitter in Different Grades of OSMF

Taste		Grade			Total	*P-value*
		I	II	III		
	Dysguesia	0 (0%)	1 (100%)	0 (0%)	1 (100%)	
Salt	Hyperguesia	1 (100%)	0 (0%)	0 (0%)	1 (100%)	0.014*
	Hypoguesia	0 (0%)	0 (0%)	3 (100%)	3 (100%)	
	Normal	11 (44%)	11 (44%)	3 (12%)	25 (100%)	
	Total	12 (40%)	12 (40%)	6 (20%)	30 (100%)	
	Dysguesia	0 (0%)	1 (100%)	0 (0%)	1 (100%)	
Bitter	Hyperguesia	1 (100%)	0 (0%)	0 (0%)	1 (100%)	
	Hypoguesia	0 (0%)	0 (0%)	3 (100%)	3 (100%)	0.014*
	Normal	11 (44%)	11 (44%)	3 (12%)	25 (100%)	
	Total	12 (40%)	12 (40%)	6 (20%)	30 (100%)	

*, P value less than 0.05 is statistically significant

Four different solutions for four basic tastes (sweet, salty, bitter and sour) were freshly prepared for gustatory testing. Sucrose for sweet (1.0 mol/l), citric acid for sour (1 mol/l), sodium chloride for salty (1.0 mol/l) and quinine hydrochloride (1.0 mol/l) for bitter were used. Before taste assessment in the individuals, the subjects were asked not to eat or drink 1-hour prior procedure and asked to rinse the mouth with water. Four different tastants were directly applied with filter paper dipped in the solution on the dorsum of the tongue, approximately for 5 seconds. Then, they were asked to identify the taste (sweet, salty, sour, bitter or tasteless) and intensity of the taste was noted. This procedure was carried out in the same way for all the four taste tastant in an individual. The tastes perceived by the subjects were recorded as hypogeusia, hypergeusia, dysgeusia, and ageusia (NHANES, 2013; Stillman et al., 2003)

*Inference*

• Hypoguesia: Diminished sensitivity of taste with elevated thresholds and reduced ability to perceive supra threshold stimuli.

• Hyperguesia: Elevated sensitivity of taste.

• Dysguesia: Distorted normal taste.

• Aguesia: Absence of taste (Abdul Khader, 2015).

*Statistical analysis*

All the findings were entered in Microsoft Excel using SPSS 20.0 software and were expressed as mean ± standard Deviation and calculated and compared using Chisquare test. The degree of freedom between variables was also observed. P value less than 0.05 indicated significant association at 5% level of significance.

## Results

In the present study, 90 subjects aged between 20 to 45 years with the mean age of 29 years were included. Study comprises of 82males and8 females. Of 30 OSMF patients, twelve subjects belonged to stage I and twelve belonged to stage II and six belonged to stage III Oral submucous fibrosis respectively. Of the 30 OSMF subjects; 23 (76.7%) normal, 06 (20.0%) hypogeusia, 1(3.3%) hypergeusia, with no subjects showing dysgeusia and ageusia to sweet taste; 3 (10.0%) hypogeusia, 25 (83.3%) normal, 1 (3.3%) dysgeusia, and 1 (3.3%) hypergeusia no subjects showing ageusia to salty taste; 10 (33.3%) hypogeusia, 19 (63.3%) normal, and 1 (2.2%) hypergeusia to sour taste;4 (10.0%) hypogeusia, 25 (83.3%) normal, 1 (3.3%) dysgeusia, and no subjects showing ageusia and hyperguesia to bitter taste (Table 1 and Table 2).

Out of the 30 control subjects, 1 (3.3%) hypogeusia to salty taste, 3 (10.0%) hypergeusia to sour taste, 2 (6.7%) hypergeusiaand1 (3.3%)dysgeusia to sweet taste and 1 (3.3%) hypogeusia to bitter taste were observed. The remaining subjects had the normal perception (90%) to the different tastants (Table 1 and Table 2).

Out of the 30 Gutka subjects, 4 (13.3%) hypogeusia to salty taste, 4 (13.3%) hypergeusia to sour taste, 1(3.3%) hyperguesia and 4(13.3%) hypoguesia to bitter taste and 4(13.3%) hypogeusia to sweet taste were observed. The remaining subjects had the normal perception (86.3%) to the different tastants (Table 1 and Table 2).

Results showed that delayed perception was noticed with sour followed by salt and sweet in OSMF patients as compared with control. Comparison of taste perception among the various tastants between the three stages of OSMF inferred 12 normal to sweet; 1 hypergeusia and 11 normal to salty taste; 1hypergeusia, 4 hypogeusia and 7 normal to sour taste, 1hyperguesia and 11normal to bitter among twelve Stage I OSMF subjects. Twelve subjects of Stage II OSMF displayed 1 hyperguesia, 5 hypogeusia, and 6 normal to sweet,1dysgeusia, and 11 normal to salty taste, 1dysguesia to bitter taste. Six Stage III OSMF subjects showed 1 hypoguesia to sweet and 5 hypoguesia to sour and 3 hypoguesia to salt taste 3 hypoguesia to bitter taste (Table 3 and 4). 

## Discussion

OSMF is a peculiar, chronic progressive, insidious, irreversible, crippling disease of the oral cavity characterized by fibrotic change and severe burning sensation with restricted opening of the mouth. The disease is characterized by blanching and stiffness of oral mucosa, trismus, and burning sensation in the mouth. It also produces hypomobilty of the soft palate and tongue, and loss of gustatory sensation (Ali et al., 2013).

Gustation is important sense as it is main sensory system that keeps check on ingested food (Zion et al., 2004). Human beings are able to perceive the four basic tastes: sweet, salt, sour and bitter and recently, a new taste umami is also incorporated. Neurologically each taste response is independent to distinct component system (Gyton, 2002). It is generally assumed that decline in taste sensitivity occur after the age of 60 years. Mojet et al., (2001) described that changes in the gustatory sensation can also occur as an aging process. According to Winkler et al., (1999) sensitivity to salty and bitter tastes declines with age. Many pathological conditions may lead to alteration in taste out of which one condition may be OSMF due to chronic chewing of areca nut which causes continuous irritation to the oral cavity and other intraoral structures.

In previous studies taste strips and electrogustometry was used for taste perception (Dyasanoor and Abdul Khader, 2016; Soni et al., 1981). In the study done by deeplakshmi et al., (2012) spatial testing followed by solutions were used for assessing taste in OSMF and controls whereas in present study only spatial testing by solutions was used for assessing taste over the dorsum of the tongue was done as many taste buds are located on dorsum of the tongue.

In the study done by Abdulkhader et al., (2015) areca nut group showed hyperguesia to bitter (35.6%) and sweet (28.9%) taste followed by hypoguesia (31.1%) to sour and normal(35.6%) to salty taste. In our study Gutka chewers showed 13.3% hypoguesia to all four tastants and 3.3% hyperguesia to bitter taste.

The results of study done by Deeplakhmi et al., (2012) showed delayed perception with sweet followed by salt, bitter and sour in OSMF subjects and in study done by Abdulkhader et al., (2015), OSMF group showed hypoguesia (62.2%) to salty taste followed by dysguesia (40%) to sour, and normal (55.6% and 48.9%) to bitter and sweet taste. In present study OSMF group showed 20% hypoguesia to sweet and 10%hypoguesia to salty and 33.3% to sour and 13.3% to bitter taste. During areca nut, chewing lot of chemicals and metals such as alkaloids, tannins, copper, iron are leached out into saliva, which in turn alters the property and composition of saliva which is the main etiological reason for OSMF which causes clinically progressive limited mouth opening, fibrosis, burning sensation (Shruthal and Alka Dive, 2015). So, among OSMF and Gutka chewers group, decreased or altered perception may be due to inflammation and infection in oral cavity that decreased blood supply and atrophy of the papillae and decrease in salivary flow rate. Decreased salivary flow rate damages the taste cell receptors in turn there is atrophy of taste buds. With increase in severity of grades of OSMF, statistical significance is seen with sour and salt taste. This may be due to, with increase in severity of OSMF there is increase in inflammation and atrophy.

In conclusion, the present study demonstrated impaired taste sensation in Gutka chewers and OSMF subjects compared to controls. Hypoguesia was observed more (33.3%) in OSMF patients when compared to gutka chewers (13.3%) so, we can conclude that taste perception is significantly affected in OSMF as well as gutka chewers, which in turn affects the balanced nutrition and quality of life of the patients and further studies should be carried out to understand about taste dysfunction in OSMF patients and its effect on malignant transformation.
